# Steroid-Associated Hip Joint Collapse in Bipedal Emus

**DOI:** 10.1371/journal.pone.0076797

**Published:** 2013-10-21

**Authors:** Li-Zhen Zheng, Zhong Liu, Ming Lei, Jiang Peng, Yi-Xin He, Xin-Hui Xie, Chi-Wai Man, Le Huang, Xin-Luan Wang, Daniel Tik-Pui Fong, De-Ming Xiao, Da-Ping Wang, Yang Chen, Jian Q. Feng, Ying Liu, Ge Zhang, Ling Qin

**Affiliations:** 1 Department of Orthopaedics & Traumatology, The Chinese University of Hong Kong, Hong Kong SAR, China; 2 Department of Orthopaedics & Traumatology, Shenzhen Second People's Hospital, Shenzhen, China; 3 Translational Medicine R&D Center, Institute of Biomedical and Health Engineering, Shenzhen Institutes of Advanced Technology, Chinese Academy of Sciences, Shenzhen, China; 4 Orthopedic Research Institute, General Hospital of Chinese People's Liberation Army, Beijing, China; 5 Baylor College of Dentistry, University of Missouri-Kansas City, Kansas City, Missouri, United States of America; 6 Department of Orthopeadics, Shenzhen Hospital of Beijing University, Shenzhen, China; 7 Department of Orthopaedics, Zhongda Hospital of Southeast University, Nanjing, China; 8 School of Chinese Medicine, Hong Kong Baptist University, Hong Kong SAR, China; INSERM U1059/LBTO, Université Jean Monnet, France

## Abstract

In this study we established a bipedal animal model of steroid-associated hip joint collapse in emus for testing potential treatment protocols to be developed for prevention of steroid-associated joint collapse in preclinical settings. Five adult male emus were treated with a steroid-associated osteonecrosis (SAON) induction protocol using combination of pulsed lipopolysaccharide (LPS) and methylprednisolone (MPS). Additional three emus were used as normal control. Post-induction, emu gait was observed, magnetic resonance imaging (MRI) was performed, and blood was collected for routine examination, including testing blood coagulation and lipid metabolism. Emus were sacrificed at week 24 post-induction, bilateral femora were collected for micro-computed tomography (micro-CT) and histological analysis. Asymmetric limping gait and abnormal MRI signals were found in steroid-treated emus. SAON was found in all emus with a joint collapse incidence of 70%. The percentage of neutrophils (Neut %) and parameters on lipid metabolism significantly increased after induction. Micro-CT revealed structure deterioration of subchondral trabecular bone. Histomorphometry showed larger fat cell fraction and size, thinning of subchondral plate and cartilage layer, smaller osteoblast perimeter percentage and less blood vessels distributed at collapsed region in SAON group as compared with the normal controls. Scanning electron microscope (SEM) showed poor mineral matrix and more osteo-lacunae outline in the collapsed region in SAON group. The combination of pulsed LPS and MPS developed in the current study was safe and effective to induce SAON and deterioration of subchondral bone in bipedal emus with subsequent femoral head collapse, a typical clinical feature observed in patients under pulsed steroid treatment. In conclusion, bipedal emus could be used as an effective preclinical experimental model to evaluate potential treatment protocols to be developed for prevention of ON-induced hip joint collapse in patients.

## Introduction

Steroid-associated osteonecrosis (SAON) is a common orthopaedic problem although steroids are initially prescribed for many non-orthopedic medical conditions, such as systemic lupus erythematosus (SLE), organ transplantation, asthma, rheumatologic arthritis (RA), and severe acute respiratory syndrome (SARS) [1]–[5]. Part of SAON patients even evolved to hip joint collapse with subsequent total joint replacement [6], [7], and its long-term durability however still remained a big challenge [8]. How to prevent accumulation of SAON lesions is the first-line strategy for avoiding joint collapse. Therefore, establishment of appropriated SAON animal models that mimic clinical etiology and even evolves to joint collapse is desirable prior to translating prevention and treatment experimental protocols into clinical validation and applications.

Up to now there is lack of ideal animal models to exam the treatment efficiency or therapeutic strategy for SAON-associated joint collapse. The limitation of the existing animal models, such as rabbit [9]–[13], rat [14]–[17], mouse [18], [19], pig [20], [21] and chicken [22] is that they fail to progress to the end-stage of SAON, i.e. structural collapse of the weight-bearing joints. With bipedality, high activity level and large enough bodyweight similar to that of human beings, ON model to be developed in emu femoral head could provide a unique opportunity to progress to human-like femoral head collapse [23], [24]. Focal cryogenic (liquid nitrogen) insults [23], [25] and alternative cooling and heating insults [26] have also been tested to induce ON in emus with femoral head collapse. However, these models are not etiology- and or pathophysiology-orientated for SAON research. Accordingly, the aim of the current study was to establish a SAON model in bipedal emus, with potentials to bone structural deterioration with subsequent femoral head collapse, a condition seen in SAON patients attributed to similar biomechanics or loading ratio imposed onto the hip joint [23], [24]. Such a model would be essential for testing strategies to be developed for potential clinical applications for prevention and treatment of steroid-associated joint collapse.

Of all available animal models, rabbits were intensively used for establishing ON model where either lipopolysaccharide (LPS) [27] or methylprednisolone (MPS) [12], [28], [29] or their combination (LPS+MPS) [11], [13] were tested. All of them showed effectiveness in ON induction, yet with varying degrees of ON lesions and mortality of animals. Based on our established SAON rabbit model with a high incidence of ON and low or no mortality that was induced by a combination of LPS and MPS [11], [30], we hypothesized that such a combination of pulsed LPS and MPS injections might also be able to induce SAON in bipedal emus with subsequent hip joint collapse.

## Materials and Methods

### Ethics Statement

The research ethics committee of Shenzhen Second Peoples' Hospital reviewed and approved the experimental protocols [Licence No. 2009–001] (Appendix S1). Both the *Guide for the Care and Use of Laboratory Animal* (1996) [31] and the ARRIVE (Animals in Research: Reporting *In Vivo* Experiments) guidelines [32] were followed.

### Animals, grouping and treatment

Eight 24 months old young adult male emus were used for this study. They were kept in Shenzhen Emu Institute and received food and water *ad libitum*. Five emus assigned to the SAON group were treated with a combination of LPS and MPS. Three emus were used as controls without receiving either LPS or MPS. The emus were euthanized by intravenous injection of overdose of pentobarbital via jugular vein at 24 weeks post injection. The details of this combined protocol were described as follows: each emu was intravenously injected with lipopolysaccharide (Escherichia coli O111:B4; Sigma-Aldrich, St. Louis, MO, USA) twice via jugular vein with 8 μg/kg body weight at an interval of 4 days from day 0. Thereafter, three injections of methylprednisolone (Pharmacia & Upjohn, Peapack, NJ, USA) with 10 mg/kg body weight were given intramuscularly at gluteus muscle at an interval of 2 days. In addition, each emu was intramuscularly injected at gluteus muscle with 40 mg Omeprazole Sodium and orally with 250 mg Amoxicillin Dispersible per day for 7 days immediately after induction to prevent potential stomach ulcers and systemic infection (Figure S1).

### Magnetic Resonance Imaging (MRI)

MRI was performed with a 1.0 T MR unit (Magnetom Harmony; Siemens, Erlangen, Germany) at baseline, week 2 and then at monthly basis on SAON induced emus for *in vivo* examination on bilateral proximal femora until 12 weeks post induction. For facilitating *in vivo* bioimaging examination, a specific posture fixture was designed to obtain a highly reproducible image during MRI scanning (Figure S2). A phased-array body coil was used for MRI scanning. Coronal turbo spin-echo fat-saturated T2-weighted images (4000 ms repetition time, 96 ms echo time) were obtained with a slice thickness of 3 mm and interslice gap of 0.3 mm from a field of view of 300 mm × 300 mm with a matrix of 320×320 pixels.

### Hematological evaluation

Blood was sampled at baseline, week 2, 4 and 8 post induction for routine blood examination and serum was prepared for examination of both coagulation and lipid metabolism. The serum parameters related to lipid metabolism, including Total Cholesterol (TC), Total Glycerin (TG), Low-Density Lipoprotein (LDL) and High-Density Lipoprotein (HDL), and the serum parameters related to coagulation, including Prothrombin Time (PT), Prothrombin Time and International Normalized Ratio (PT/INR), Fibrinogen (FBG) and Thrombin Time (TT), were tested using standard clinical laboratory protocols for blood chemistry in Shenzhen Second Peoples' Hospital.

### Gait observation

Gait of emus with SAON was observed regularly after induction by recording abnormal gait pattern using a video camera. Normal gait of the control emus was also recorded for comparison (Video S1 and S2). Emus walked or ran on a 20-meter walkway, and the sagittal plane motion was videotaped at 300 Hz by a high-speed video camera (Casio EX-F1, Japan). The videos were analyzed by a motion analysis system (Kwon3D XP, Korea) to obtain the included ankle joint angle during a gait stride cycle with respect to the time presented in term of percentage stride. Gait pattern was defined abnormal when there was a deviation of the included ankle joint kinematics (more than 20 degrees at any time) or the proportion of the stance and swing time (more than 10% deviation from control case).

### Micro-CT scanning and analysis

Bilateral proximal femora from both control group and SAON group were sampled and fixed in 10% buffered neutral formalin solution for 10 days, and then soaked in 70% ethyl alcohol for measurement of trabecular morphology within and around ON lesions. In brief, the proximal femur was scanned using a high-resolution peripheral CT (HR-pQCT) (Xtreme CT, Scanco Medical, Brüttisellen, Switzerland) at a voltage of 70 kV and a current of 114 uA, with entire scan length of 40 mm in a spatial resolution of 40 μm used for animal experimental studies [33], [34]. After the initial scanning, 2-dimentiaonal (2–D) images were realigned in the Z-axis along the direction of femoral neck for further evaluation. For separating the signals of the mineralized tissue from the background signal, noise was removed using a low-pass Gaussian filter (Sigma = 2.5, Support = 2) and mineralized tissue was then defined at a threshold of 85. 3–D structures of entire femoral heads were then reconstructed. The collapse was identified when fracture and/or clear deformation appeared in femoral head. A 10 mm ×10 mm ×8 mm region in the centre of femoral head was defined as the region of interest (ROI) for analysis and comparison. ROI was defined within subchondral region centered in the collapsed region or the corresponding region of the non-collapsed femoral heads, where the largest thickness of this ROI was 1/3 diameter of the femoral head. Bone mineral density (BMD), bone tissue volume fraction (BV/TV), trabecular number (Tb. N), trabecular thickness (Tb. Th) and trabecular separation (Tb. Sp) in the ROI were measured separately by the workstation with the built-in HR-pQCT software.

### Histology

After micro-CT scanning, femoral heads were sawed in half longitudinally along the coronal plane. Halves were decalcified and embedded in paraffin; and halves were embedded in Methyl methacrylate (MMA) without decalcification.

#### 1. Decalcified sections

For paraffin embedded samples, sections were sliced along the coronal plane for hematoxylin-eosin (H&E) and fast green and safranin O staining, respectively. A microscope imaging system (Leica Q500MC; Leica Micro-systems, Wetzlar, Germany) was used to digitalize the histological sections for histomorphometric evaluation. 1) Examination of ON: The entire area of each H&E stained section was examined for the presence of ON with the established criteria, i.e. diffuse presence of empty lacunae or pyknotic nuclei of osteocytes in the trabeculae, accompanied by surrounding necrotic bone marrow [13], [28]. The femoral head with at least one ON lesion was considered as ON+, while that with no ON lesion was considered as ON- [11], [35]. 2) Quantification of fat cells in bone marrow: Fat cells were quantified for both average of fat cell size and fat cell area fraction. The total bone marrow in subchondral bone was 200 fold magnified and captured. The fat cells were manually traced and then quantified. The fat cell size was interpreted by Fetet's diameter, i.e. the longest distance between any two points along a region of interest (ROI) boundary [11]. The fat cell area fraction was defined as total marrow fat cell area normalized by total marrow tissue area [35]. 3) Histomorphometry of the subchondral plate: the thickness of subchondral bone plate of the femoral head was measured on the 50× images. At least 10 views per section were randomly selected for measuring. The thickness was examined by measuring point-to-point distance from the top of the calcified cartilage to the deep surface of the subchondral bone plate [36]. 4) Examination of articular cartilage: The average thickness of cartilage were examined by the total area of cartilage divided by the length of cartilage band measured from the manually traced region on the entire frontal sections for evaluation under 200× magnification. The thickness of cartilage at the collapsed region or the corresponding region of the non-collapsed femoral head was also examined. The proteoglycans content was quantified by measuring the thickness of safranin O stained articular cartilage. 5) Calculation of osteoblasts: The osteoblast perimeter percentage (%Ob.Pm) was calculated as the ratio of the total perimeter of the trabecular surface covered by osteoblasts to the whole perimeter of the trabecular surface [37]. 6) Blood vessel quantification: The number of blood vessels within the collapsed region or the corresponding region of the non-collapsed femoral head was quantified on H&E sections [38].

#### 2. Undecalcified sections

The MMA embedded femoral heads were sectioned along the sawed plane using a diamond saw (Isomet, Buehler). The cut surface was polished on a soft cloth rotating wheel [39]. The surfaces were acid-etched with 37% phosphoric acid for 2–10 seconds, followed by 5% sodium hypochlorite for 20 minutes. The samples were then sputter-coated with gold and palladium, as described previously [40], [41] and examined for bone matrix and features of osteocytes in the scanning electron microscope (SEM) (JSM-6300, JEOL, Japan).

### Statistical analysis

Statistical power was set >0.8 and the Type I error probability was set <0.05 for calculating sample size (n = 5) using PS (Power and Sample Size Calculations Version 3.0) for establishing SAON model based on our previous rabbit model with a SAON incidence of 93% [11].

The incidence of collapse was defined as the number of collapsed hips divided by total number of hips in each group. The incidence of SAON was defined as the number of SAON emus divided by total number of emus in each group, and analyzed with fisher's exact test. The serum parameters were expressed as Mean ± SD, and analyzed by one way analysis of variance (ANOVA) with a post hoc Bonferroni's multiple comparison test to compare the differences between every time point and baseline. Micro-CT and histomorphometry data were expressed as Mean ± SD, and analyzed with Mann Whitney test to compare the differences between the control group and SAON group. SPSS 10.0 was used. The significance for comparison was set at *p*<0.05.

## Results

### MRI findings

**Figure 1:**
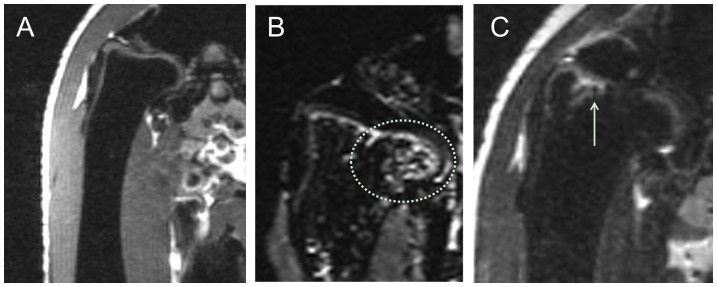
Representative T2-weighted coronal MRI images of an emu in SAON group. (A) baseline before SAON-induction; (B) at week 2 post SAON-induction, showing edema (dotted circle) in the proximal femur; and (C) at week 12 post SAON-induction, showing decreased edema (arrow) in the proximal femur. No collapse was found in the first 12 weeks after LPS-MPS induction.

In T2 weighed MRI images, intense signals of edema in the proximal femur were found at week 2 post-induction when compared to the baseline. The edema signals in proximal femur decreased in T2 weighed MRI image at week 12 post induction (Figure 1). No collapse was found from MRI images in the first 12 weeks after induction.

### Hematological evaluation

**Figure 2:**
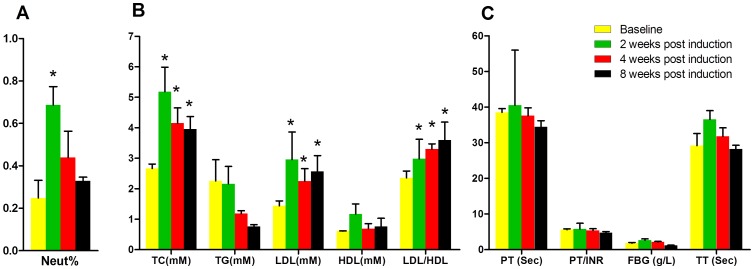
Hematological evaluation of emus in SAON group. (A) Time course changes in percentage of neutrophils (Neut %) in blood: Neut% was significantly increased at 2 weeks post SAON-induction (*: *p*<0.05, compared with baseline, n = 5). (B) Time course changes in serum parameters related to lipid metabolism: TC significantly increased at each time point post SAON-induction. LDL and the ratio between LDL and HDL also significantly increased at each time point post SAON-induction (*: *p*<0.05, compared with baseline, n = 5). (C) Time course changes in serum parameters related to coagulation: no significant difference was found in coagulation-related parameters after induction as compared with baseline.

The results of routine blood examination showed abnormal increase in percentage of neutrophils (Neut %) at 2 weeks post induction (Figure 2A). For the time course changes in serum parameters related to lipid metabolism, TC was significantly increased at each time point post-induction, and ratio between LDL and HDL as well as LDL was also significantly increased at each time point post-injection (*p*<0.05 for all) while TG did not show significant increase post-induction (Figure 2B). However, as compared with baseline, no significant difference was found for the time course changes in serum parameters related to coagulation post-induction (Figure 2C).

### Gait patterns

**Figure 3:**
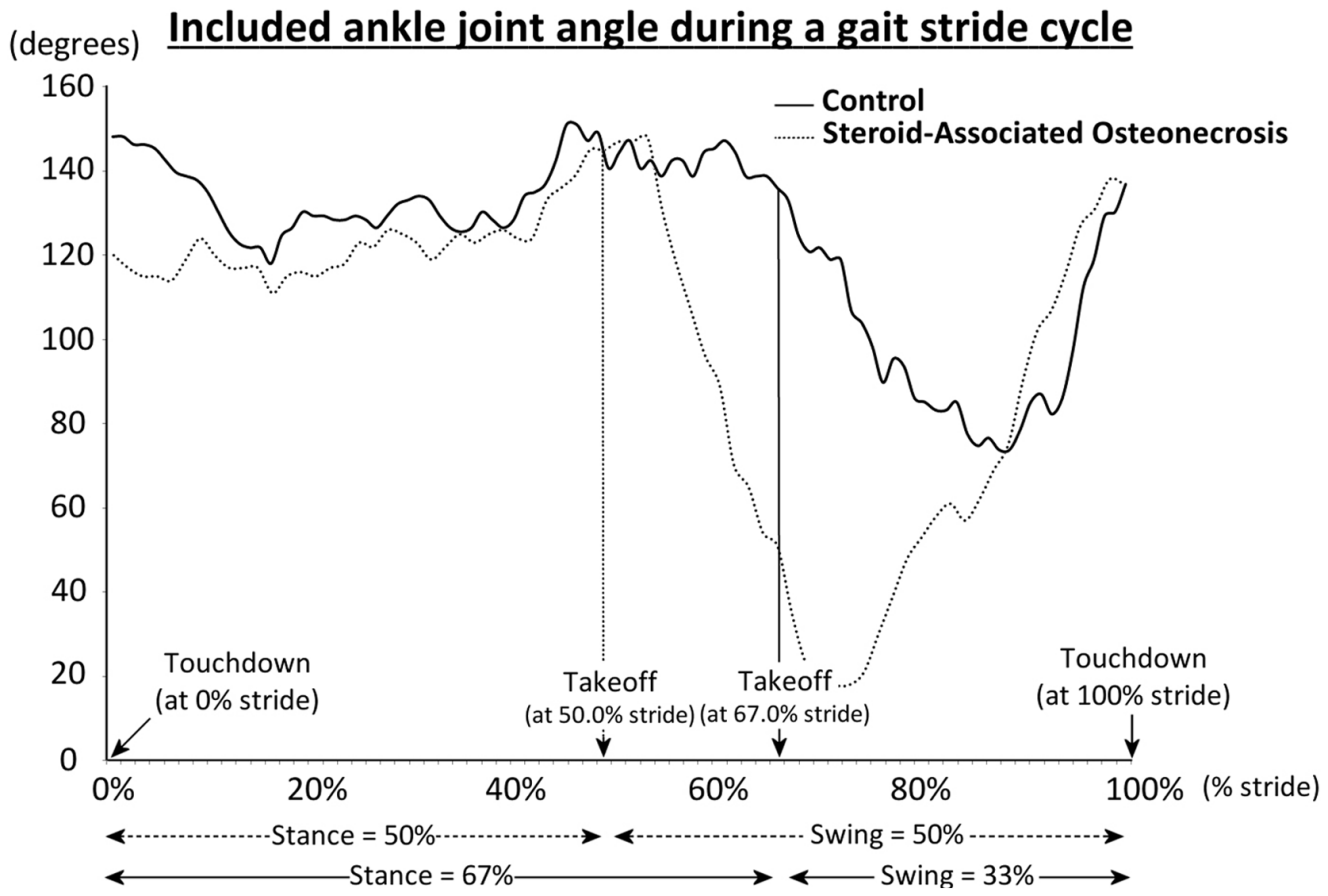
High-speed video gait analysis for the included ankle joint angle during a gait stride cycle compared between a representative normal emu and an emu with SAON-induced hip joint collapse (confirmed after its sacrifice). The major difference was shown in the ankle joint during takeoff at 67.0% stride.

All emus were observed to be less active but with normal gait in the next day after the first LPS injection; while an asymmetric limping gait pattern was firstly observed at week 12 post induction during loading and unloading gait cycle (Figure 3). In the early post-induction phase the asymmetric limping gait was only observable when the emus were running. With time the asymmetric limping gait was observed when emus were walking; closing to week 24 post induction some of the emus were observed no more active and kept sitting most of the time.

### SAON at femoral head and its collapse

**Figure 4:**
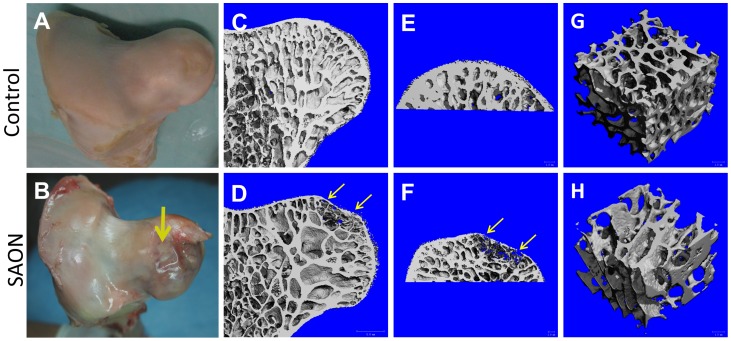
Gross observation of femora head and its micro-CT analysis compared between the control and SAON emus. (A) and (B): representative femoral head in the control group and SAON group, respectively with collapsed femoral head in SAON group (yellow arrow); (C) and (D): representative microCT images of normal femoral head in the control group and of collapsed femoral head in SAON group, respectively; (E) and (F): representative microCT images of subchondral bone in the control group and SAON group, respectively; (G) and (H): representative microCT images of trabecular bone in the central part of the femoral head in the control group and SAON group, respectively. Yellow arrow: collapsed region.

No emus died during the experiment period over 24 weeks. All five emus in SAON group developed edema, an early stage sign of ON at bilateral hip based on MRI observations as described above, in part also shown by gross morphology after sample harvesting (Figure 4A and B), and confirmed histologically as described below. The incidence of hip joint collapse was 70% (7 out of 10 hips from 5 emus) found in the SAON group, including 3 emus with bilateral collapse, 1 emu with unilateral collapse, and 1 without hip joint collapse (Figure 4A-F). There was no collapse of the hip found in control group.

### Micro-CT analysis

Micro-CT analysis was performed for the trabecular bone microarchitecture in the subchondral region (Figure 4E and F) and in the center of the femoral head (Figure 4G and H), respectively. Within the subchondral region, the BMD, BV/TV, Tb. N and Tb. Th in the SAON group were found significantly lower than those in the control group (*p*<0.05 for all), while the Tb. Sp in the SAON group was significantly higher than that in the control group (*p*<0.05) (Table 1). However, no statistically significant difference was found for all micro-CT indices of trabecular bone in the centre of the femoral head between the control and SAON group (Table 1).

**Table 1:** Micro-CT evaluation of the control group and SAON group.

	Control (n = 3, from 6 bilateral hips)			SAON (n = 5, from 10 bilateral hips)		
**The central part of the femoral head**						
BMD (mg/cm^3^)	169.767	±	29.174	171.301	±	42.791
BV/TV (%)	27.37	±	4.62	28.45	±	5.15
Tb.N (1/mm)	0.810	±	0.071	0.814	±	0.116
Tb.Th (mm)	0.399	±	0.034	0.420	±	0.0934
Tb.Sp (mm)	1.234	±	0.077	1.235	±	0.131
**The subchondral bone region**						
BMD (mg/cm^3^)	210.278	±	20.571	152.906	±	27.509 *
BV/TV (%)	47.85	±	3.99	33.18	±	3.76 *
Tb.N (1/mm)	1.261	±	0.132	1.148	±	0.076 *
Tb.Th (mm)	0.502	±	0.038	0.407	±	0.061 *
Tb.Sp (mm)	0.882	±	0.087	1.023	±	0.016 *

Mean ± SD, *: P<0.05.

### Histological evaluation

**Figure 5:**
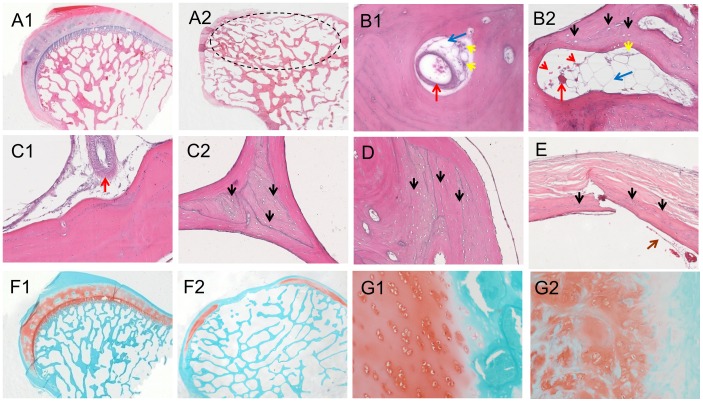
Representative histological images. H&E stained femoral heads of the control group (A1), and SAON group (A2) (dotted circle: collapsed region). Subchondral bone in the control group (B1), and in SAON group (B2) with empty lacunae (black arrows) in bone matrix, increased size and number of fat cells in bone marrow (blue arrows) and decreased number of mononuclear cells (yellow arrows) compressed blood vessel (red arrows) with effused blood cells (red arrowheads) when compared with that of the control group. Representative histological images at different regions of the femoral head: B: beneath the articular cartilage; C: central of the femoral head of normal group (B1, C1) and SAON group (B2, C2); and D: near to the femoral neck; E: fractured subchondral trabecular bone from a collapsed femoral head of SAON group, showing typical histological evidences of osteonecrosis, i.e., numerous empty lacunae or pyknotic nuclei of osteocytes (black arrows) and scattered bone marrow tissue (brown arrows). Safranin O stained femoral head in the control group (F1) and in SAON group (F2). Normal cartilage in the control group (F1) and pathological changes of articular cartilage in SAON group (F2). (10× for A1, A2, F1, F2; 200× for B1, B2, C1, C2, D, E, G1, G2).

**Figure 6:**
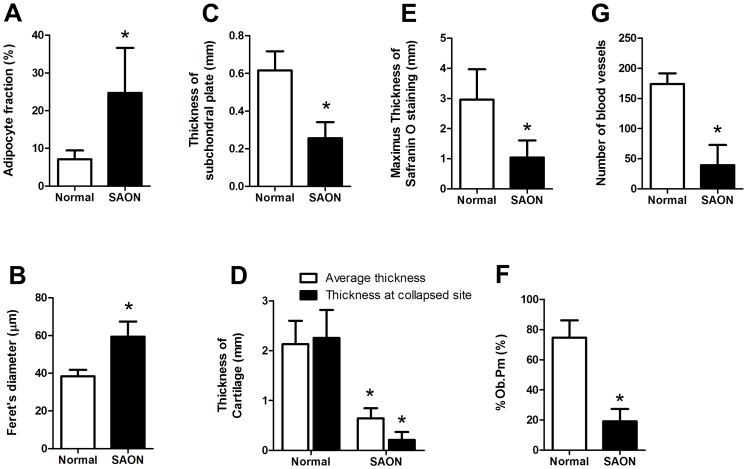
Histomorphometrical analysis of emu femoral head. In SAON group there were significantly higher adipocyte fraction (A), higher adipocyte size (B), thinner subchondral plate (C), thinner cartilage (D), thinner safranin O staining (E), smaller % Ob. Pm (F) and significant more blood vessels per slides at collapsed region or the corresponding region of the non-collapsed femoral head (G). Mean ± SD, n = 5 (10 bilateral hips) for SAON group and n = 3 (6 bilateral hips) for the control group, *: *p*<0.05).

Gross coronal view of H&E stained femoral head in the control group showed well-arranged trabecular bone supporting the subchondral plate and the articular cartilage (Figure 5A1); while the collapsed femoral head in SAON group was lack of vertical arranged trabecular bone to support the subchondral plate and the articular cartilage, and bone fracture shown at collapsed site (Figure 5A2). Though 70% of 10 hips from 5 emus in the SAON group developed hip collapse, 100% of hips from all emus in the SAON group developed ON at bilateral hips as confirmed histologically while as expected no ON lesion was found in the control emus (p<0.05) (Figure 5B, C, D, E). Osteonecrosis was distributed in whole femoral head, including subchondral bone (Figure 5B2, E), middle of the femoral head (Figure 5C2) and femoral neck (Figure 5D), with numerous empty lacunae in the trabeculae and marrow tissue degenerated. Compared with the control group, the marrow fat cell size (Fetet's diameter of fat cell) and fat cell area fraction in the SAON group were increased significantly with decreased number of mononuclear cells (p<0.05 for both, Figure 5B and Figure 6A, B). The thickness of subchondral plate of femoral head of SAON emus was decreased significantly (*p*<0.05, Figure 6C). The articular cartilage of SAON emus also showed pathological alteration with significantly thinner thickness (*p*<0.05, Figure 5F, G and Figure 6D). The proteoglycans content as interpreted by maximum thickness of safranin O staining was decreased significantly in SAON group (*p*<0.05, Figure 6E), with osteonecrosis located at the collapsed region (Figure 5E). The osteoblasts perimeter percentage (% OB. Pm) of the SAON group was significantly lower than those in the control group (*p*<0.05, Figure 6F). The blood vessels at subchondral region of SAON group were surrounded with enlarged and compacted arranged fat cells, with some blood cells effused out of the vessel (Figure 5B). At the collapsed region or the corresponding region of the non-collapsed femoral head, the number of blood vessels of SAON group was significantly less than that of the normal group (*p*<0.05, Figure 6G).

**Figure 7:**
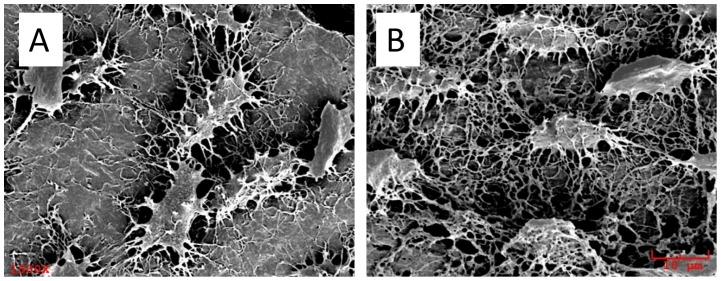
Representative/MMA-casted SEM images of the subchondral bone. A: in normal control, osteocytes are found on the solid bone matrix without obvious removal after acid processing; B: in the collapsed femur head region, osteocytes are presented without bone matrix surrounding, i.e. bone matrix removed after acid processing. (1500×).

SEM images showed that in the collapsed region there was no osteo-like structure; instead, there were more osteo-lacunae outline, with more removal of bone mineral or less matrix; and that in the normal control, there was osteon-like structure and there were few osteo-lacunae outline with much solid bone matrix (Figure 7).

## Discussion

Using a combined pulsed LPS and MPS induction protocol previously established for SAON quadrupedal rabbits [11], [13], the present study established a SAON model in bipedal emus characterized with subchondral bone deterioration and hip joint collapse, an experimental model mimicking human ON often developed at hip joint with femoral head collapse.

As bioimaging evidence, we used MRI to evaluate *in vivo* alteration of MRI signals in the first 12 weeks post induction as MRI could diagnose early-stage ON, even presymptomatically [42]. Abnormal signals were firstly detected at week 2 post-induction, showing large scales of edema at the proximal femora. This phenomena is similar to clinical ON where the bone marrow edema was found from MRI earlier than either formation of the necrotic lesion or the collapse of the necrotic fragment [43]. However, MRI of emu hips merely shows bone marrow edema without typical band pattern shown in initial MRI signaling of ON patients [44], [45]. This could be explained by the differences in anatomy and physiology of human (mammals) and emu (aves), such as that the bone marrow of emu hip mainly presented in subchondral bone in a shell-shaped region and there was hollow structure in emu's bone marrow cavity at both femoral neck and head. Irrespective such differences, there were common structural features, i.e. ON lesion and hip joint collapse found in SAON emus and this would provide a platform, i.e. a large bipedal animal model for testing biomaterials developed for bone defect repair, including for that after surgical core-decompression in hip joints, a condition indicated for patients with early stage of ON.

For functional evidence, asymmetric limping gait was observed at week 12 post-induction, suggesting that emus might suffer from joint pain caused by the ON lesion formation and structural damage confirm histologically after harvesting the samples for detailed analysis. This symptom was also common in patients at early stage of ON [46]. The asymmetric limping gait shown in bipedal emus was similar to the low limb dysfunction in SAON patients with subchondral lesions [47].

For biochemical evidence, the current study suggested hyperlipidemia occurred after SAON induction, which was evidenced by a significant increase in TC at all examined time point post-induction. In addition, abnormal higher lipid transportation to the peripheral tissues also occurred after induction, as evidenced by a significant increase in LDL/HDL, which was consistent with the results found in both SAON rabbit model [9], [11] and ON patients [48]. However, there was no significant difference in coagulation-related parameters after induction as compared with baseline, while hypercoagulable and hypofibrinolysis state were reported in SAON rabbit model [9], [11] and SARS ON patients [49]. This inconsistency suggested that the pathogenesis of SAON in emu might be explained by the dominant disorder in lipid metabolism.

Histological and histomorphometric analysis was performed in three ROIs of the femoral head, including the articular cartilage, subchondral bone region and the central part of the femoral head. Under higher magnification of the subchondral bone region where edema was shown in MRI images, remarkable necrotic changes were observed in emu after induction, including 1) avascular zone at load bearing region, referring to the blood vessels quantification within the collapsed region; 2) extravascular adipogenesis (fat cell enlargement); 3) apoptosis of osteocytes in regions next to the marrow region packed with fat cells; and 4) thinning of subchondral bone, poor quality of bone matrix and more osteo-lacunae that weakens the mechanical integrity of the femoral head, which could also be weakened by the necrosis and thinning of articular cartilage. These were essential indices of later joint collapse, typically seen in SAON patients [50], [51] that were substantiated in our histomorphometric analysis. On the contrary, our micro-CT evaluation for the central region of the femoral head, where no bone structural collapse was demonstrated, implied that the pulsed LPS-MPS induction protocol did not result in general osteoporosis as no difference in boney structural and BMD was found between SAON emus and normal controls in regions away from the subchondral bone. This finding was also similar to a bone densitometry study in SARS patients who underwent pulsed steroid treatment in Hong Kong [52]. No obvious osteoporosis found in SAON patients with joint collapse or animals were mainly explained by short-term effects of pulsed corticosteroid administration that resulted in local damage of vasculature in the subchondral bone region and impairment of bone marrow mononuclear cells, which triggered up-regulation of osteoclastic activities and inadequate bone formation known as destructive repair [1], [6], [10], [34], [53], [54]. Yet, long-term steroid administration was known to be able to induce secondary osteoporosis in patients [55] or animals [14].

In spite of the presence of difference in hip anatomy, the similarity in biomechanics of the hip joint with regard to proportion of joint loading between human and bipedal emu was well characterized by Iowa Biomechanics Research Group [56], [57]. The contact stress of femoral head was reported with a maximum hip contact force of approximately 5.5 times of body weight in emus, directed axially along its femoral neck, which was moderately larger than that of humans [56] and the contact stress magnitude and the sites of habitual loading on the femoral head was also comparable between emu hip and the human hip [57]. That the joint biomechanics plays a crucial role for NO-induced hip joint collapse was also well supported by findings obtained from bipedal and quadrupedal animals. In fact, the ON-induction protocol with a combination of LPS and MPS successfully induced SAON in quadruped animals, yet without resulting in subchondral collapse [11], although inadequate repair was also demonstrated in quadruped SAON animals [35]. Besides emu study, another report showed that the chicken was also bipedal animal suitable for building up SAON animal model [22], yet their induction protocol did not result in hip joint collapse. In order to mimic ON and hip joint collapse, we tested large bipedal emus not because of its similarity in hip joint biomechanics to that of human but mainly that emu hip was sizeable to perform core decompression and testing porous scaffold biomaterials developed for potential clinical applications [58]–[60].

The SAON induction protocol tested in the present study successfully induced hip joint collapse in large bipedal emus. The occurrence of hip joint collapse in emus was apparent more (7 out of 10 hips from 5 emus) than clinical data, such as 32.7% SAON incidence reported by Li ZR and co-workers in their well-documented clinical study in SARS patients who were treated with a high dose of corticosteroid for life-saving [61]. As SAON incidence is often dose-dependent and the purpose of the current preclinical study is to establish a SAON bipedal large animal model with high incidence of ON and hip joint collapse, we tested much higher dose of MPS as compared with clinical recommended dose for SARS patients [61]. Differ to human situation where only MPS was used, we used both LPS and MPS pulsed treatment, where LPS served the purpose to mimic clinical conditions, i.e. disease-related tissue inflammation [11], [27], in addition to higher mechanical loading imposed to the hip joint in emus where emu hip joint share 65–70% of the body weight as compared with around 60% of body weight in human [56].

In present emu study, we selected two LPS injections with 4 days interval that showed safe and effective to induce SAON. Neut % increased 2 weeks post induction and decreased gradually to baseline level from 2 weeks to 8 weeks post induction which indicated inflammation reaction induced by LPS. No typical clinical Shwartzman reaction was observed in emus as we did not systemically study the non-skeletal tissue thrombosis or reticuloendothelial blockage for comparison with that in SAON patients. It would be of interest to study in details the differences in physical or immune responses between human and birds in SAON in future. In our previous study, we established SAON in rabbit with 1×LPS (10 µg/kg) +3×MPS (20mg/kg) [11]. As emu was estimated with similar body surface area to that of human, we calculated the current experimental dose based on the conventional human-rabbit dose conversion. However, the dose for the initial experiment for emu was 6.67 mg/kg. As we found that emus could tolerate larger dosage from our pilot study, we increased MPS dose to 10 mg/kg for the current study at a time interval of 2 days for avoiding potential side-effect of MPS injection. Specific design for studying dosing effects (amount and frequency of LPS and MPS treatment) would also be of interests for further studies.

The limitation of the current experimental study is that we are not able to delineate if the cause of joint collapse is attributed to SAON and/or cartilage and subchondral bone thinning as we did not observe typical destructive repair by uncoupling of osteoclasts and osteoblasts as well as extensive local fibrosis found within necrotic regions reported in both quadrupedal rabbit model [35] or patients [62], [63]. Future studies shall be designed to monitor such pathological changes at various time points after ON-induction. Larger sample size would also be appreciated although our findings did reach statistical significance where we estimated sample size using PS (Power and Sample Size Calculations Version 3.0) for establishing our current emu SAON model based on our previous rabbit model with a SAON incidence of 93% [11].

In conclusion, this was the first experimental study to confirm that a combined injection protocol of pulsed LPS and MPS was able to induce ON and deterioration of subchondral bone microarchitecture in bipedal emus, with subsequent femoral head collapse. The establishment of this bipedal emu model with hip joint collapse provided a platform for evaluation of potential treatment protocols to be developed for prevention of steroid-associated hip joint collapse.

## Supporting Information

Figure S1 **SAON induction in bipedal emus.** A: Intravenous injection of LPS; B: Intramuscular injection of MPS; C: General conditions of emus are normal post SAON-injection of lipopolysaccharide and methylprednisolone using the current SAON induction protocol.
(TIF)

Figure S2 **A specific posture fixture is developed for in vivo bio-imaging examination of emu hips to obtain highly reproducible images.** A: Firstly, the emu is maintained at left lateral lying position, which fits the size of MRI examination bed; B: the bilateral lower limbs are kept parallel by inserting a piece of shaped foam board between the two legs in the custom-made posture fixture, which reduces variation in positioning during the repeated in vivo radiographic examinations; C: Emus are ready for MRI scanning.
(TIF)

Appendix S1 **Animal Ethics obtained from the Research Ethics Committee of Shenzhen Second Peoples' Hospital [Licence No. 2009–001] (only given in Chinese).**
(TIF)

Video S1 **Abnormal gait of SAON treated emu.**
(MPG)

Video S2 **Normal gait of normal control emu.**
(MPG)
