# Supplementary figures and images for: Steroid-Associated Hip Joint Collapse in Bipedal Emus
Source: PLoS One. 2013 Oct 21;8(10):e76797. doi: 10.1371/journal.pone.0076797 (PMC3804596; doi:10.1371/journal.pone.0076797)

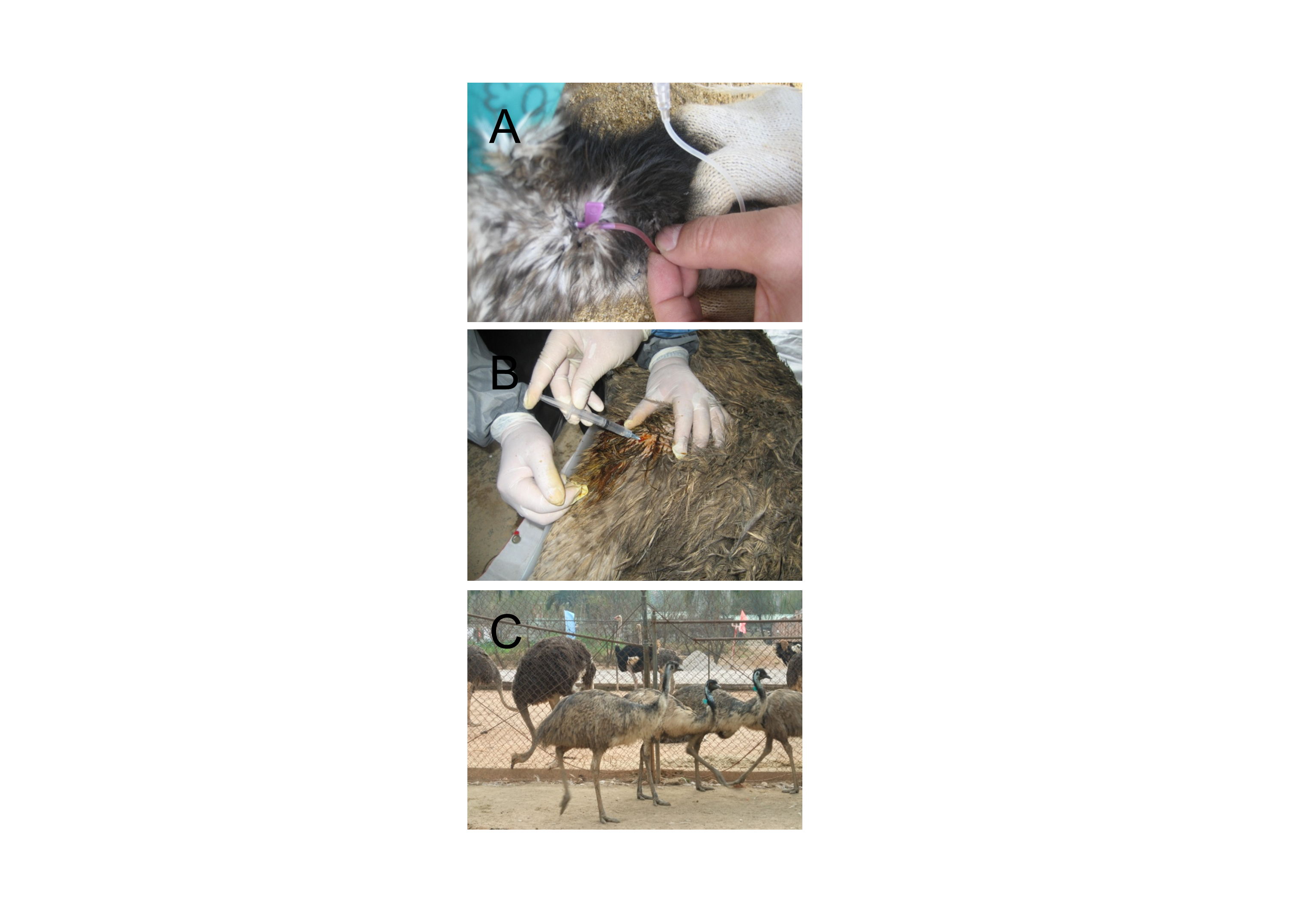

Supplement: Figure S1 — SAON induction in bipedal emus. A: Intravenous injection of LPS; B: Intramuscular injection of MPS; C: General conditions of emus are normal post SAON-injection of lipopolysaccharide and methylprednisolone using the current SAON induction protocol. (TIF) [file pone.0076797.s001.tif]

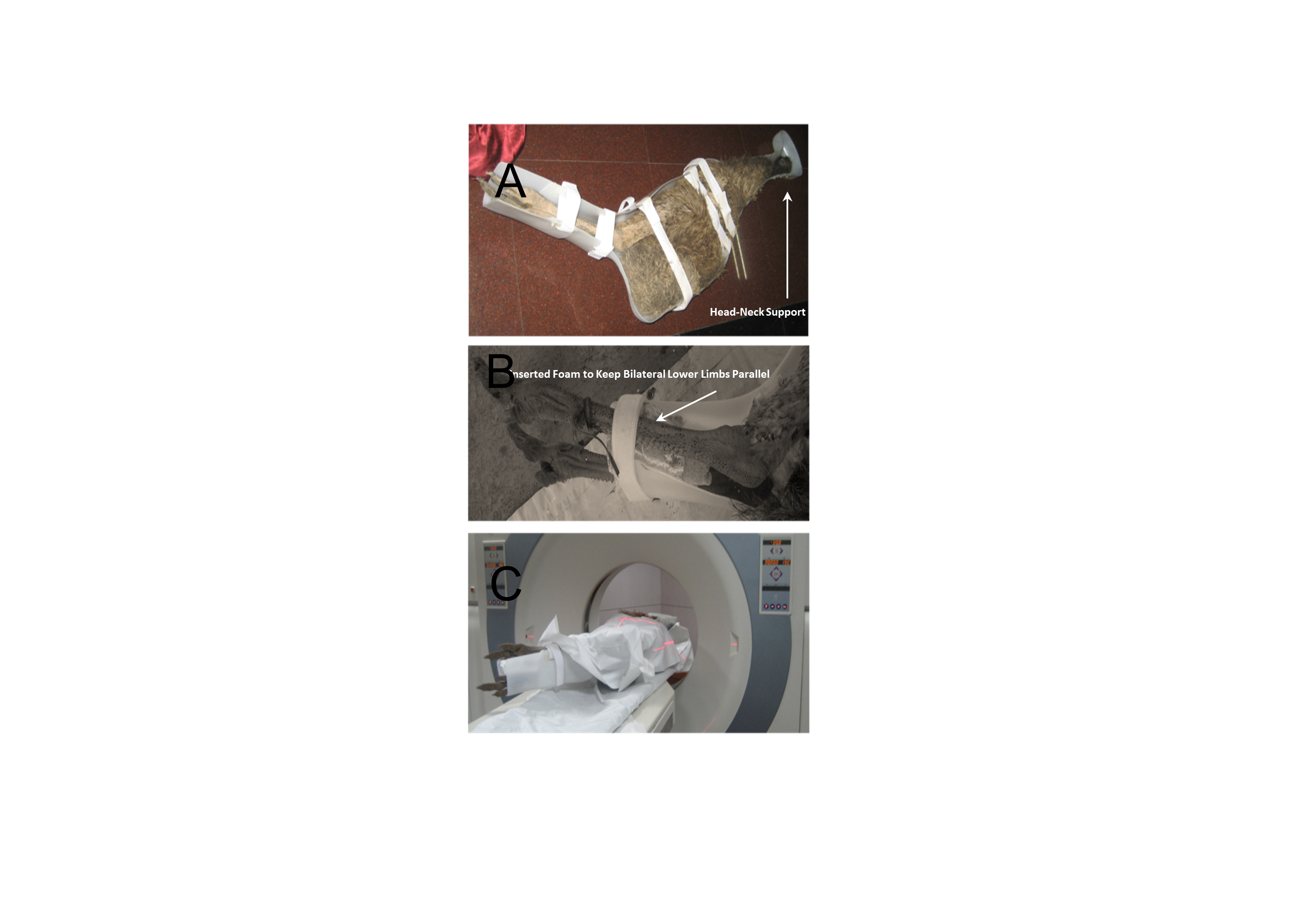

Supplement: Figure S2 — A specific posture fixture is developed for in vivo bio-imaging examination of emu hips to obtain highly reproducible images. A: Firstly, the emu is maintained at left lateral lying position, which fits the size of MRI examination bed; B: the bilateral lower limbs are kept parallel by inserting a piece of shaped foam board between the two legs in the custom-made posture fixture, which reduces variation in positioning during the repeated in vivo radiographic examinations; C: Emus are ready for MRI scanning. (TIF) [file pone.0076797.s002.tif]

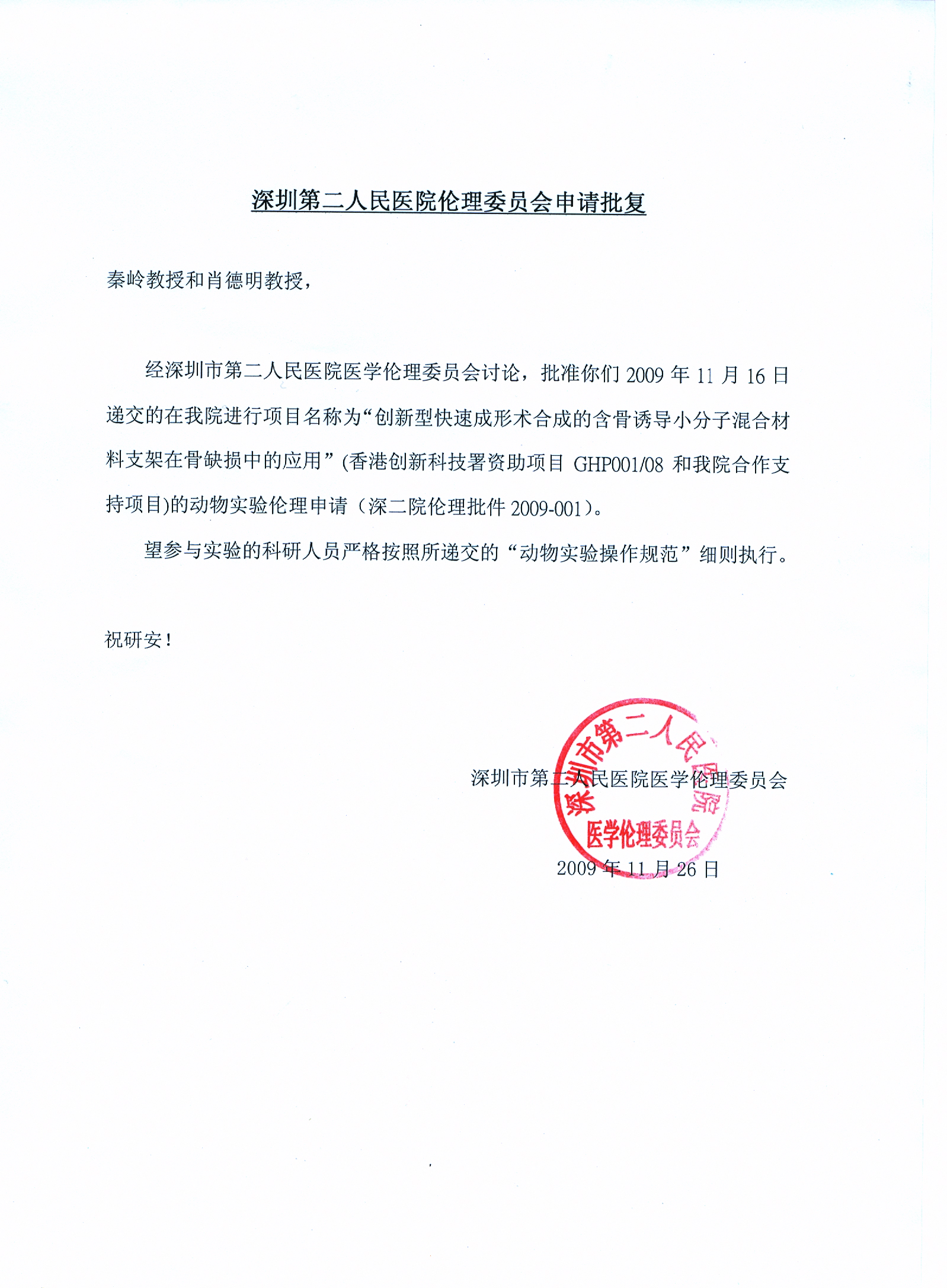

Supplement: Appendix S1 — Animal Ethics obtained from the Research Ethics Committee of Shenzhen Second Peoples' Hospital [Licence No. 2009–001] (only given in Chinese). (TIF) [file pone.0076797.s003.tif]
